# Temperature-Program Assisted Synthesis of Novel Z-Scheme CuBi_2_O_4_/*β*-Bi_2_O_3_ Composite with Enhanced Visible Light Photocatalytic Performance

**DOI:** 10.3390/nano8080579

**Published:** 2018-07-28

**Authors:** Xiaojuan Chen, Ning Li, Runliang Zhu, Shuai Li, Chunmo Yu, Wei Xia, Song Xu, Xin Chen

**Affiliations:** 1 School of Environment and Chemical Engineering, Foshan University, Foshan 528000, China; xjchen0218@163.com (X.C.); fky0322017@126.com (S.L.); cym0171@126.com (C.Y.); lining159.com@163.com (W.X.); xusong018@163.com (S.X.); fschenxin@163.com (X.C.); 2 School of Marine Sciences, Sun Yat-sen University, Guangzhou 510275, China; 3 Guangdong Provincial Key Laboratory of Mineral Physics and Materials, Guangzhou Institute of Geochemistry, Guangzhou 510640, China; zhurl@gig.ac.cn; 4 CAS Key Laboratory of Mineralogy and Metallogeny, Guangzhou Institute of Geochemistry, Chinese Academy of Sciences, Guangzhou 510640, China

**Keywords:** CuBi_2_O_4_/*β*-Bi_2_O_3_, temperature programmed, semiconductors, Z-Scheme, visible light, organics

## Abstract

Novel Z-Scheme CuBi_2_O_4_/*β*-Bi_2_O_3_ composite photocatalysts with different mass ratios and calcination temperatures were firstly synthesized by the hydrothermal method following a temperature-programmed process. The morphology, crystal structure, and light absorption properties of the as-prepared samples were systematically characterized, and the composites exhibited enhanced photocatalytic activity toward diclofenac sodium (DS) degradation compared with CuBi_2_O_4_ and *β*-Bi_2_O_3_ under visible light irradiation. The optimal photocatalytic efficiency of the composite, achieved at the mass ratio of CuBi_2_O_4_ and *β*-Bi_2_O_3_ of 1:2.25 and the calcination temperature of 600 °C is 92.17%. After the seventh recycling of the composite, the degradation of DS can still reach 82.95%. The enhanced photocatalytic activity of CuBi_2_O_4_/*β*-Bi_2_O_3_ is closely related to OH^•^, *h*^+^ and O_2_^•−^, and the photocatalytic mechanism of CuBi_2_O_4_/*β*-Bi_2_O_3_ can be explained by the Z-Scheme theory.

## 1. Introduction

Diclofenac sodium (DS) is a very effective anti-inflammatory drug widely used in many countries [1,2]. Due to the poor absorbability of organisms to DS, most of the DS will be released into the environment after it is taken [1]. However, long-term exposure to a DS-polluted environment will cause serious risk to ecosystems and human health [3,4]. Therefore, effective degradation technologies for DS should be explored to control its persistent contamination.

Semiconductor photocatalytic technology has aroused widespread interest in organic pollution control [5,6,7,8] due to its advantages of rich catalysts, mild reaction conditions, fast reaction speed, and no secondary pollution. In past decades, titanium dioxide (TiO_2_) has attracted considerable attention for its low cost and chemical inertness [8,9,10]. However, the large band gap of TiO_2_, which is approximately 3.2 eV, limits its absorption of sunlight to the ultraviolet (UV) region [10,11]. Because the visible light occupies 43% of sunlight, whereas UV light occupies only 4%, the development of visible-light responsive semiconductor photocatalysts is becoming a research hotspot [12,13,14,15].

CuBi_2_O_4_ possesses a strong visible light response, excellent chemical stability, high conduction band position, and strong reducing ability [16,17,18]. However, the single CuBi_2_O_4_ exhibits poor photocatalytic performance [17]. Some related studies suggest its activity could be improved through the synergistic effect with other metal oxide semiconductors [17,19,20]. Bi_2_O_3_, a visible light-responsive semiconductor material, is regarded as a promising photocatalyst for organic pollutant degradation [21,22]. This is mainly due to that the energy band gap (in the range from 2.4 to 3.2) of Bi_2_O_3_ can be tuned by employing different synthesis techniques, such as sol gel, hydrothermal/solvothermal and solid-state decomposition methods [21,23,24]. *β*-Bi_2_O_3_, as one of the six different polymorphs of Bi_2_O_3_ (i.e., *α*-, *β*-, *γ*-, *δ*-, *ω*- and *ε*-polymorph), exhibits a narrower band gap with the strongest light-absorption ability than other phases [23,25,26,27]. Yet the photocatalytic activity of pure *β*-Bi_2_O_3_ is still far from satisfactory for the fast recombination of photogenerated charges [26,28].

In theory, CuBi_2_O_4_ coupling with *β*-Bi_2_O_3_ could form the hybrid composite via matching the band structure of each other with significantly enhanced photocatalytic activity. Nevertheless, the research on the temperature-programmed synthesis and photocatalytic application of a CuBi_2_O_4_/*β*-Bi_2_O_3_ system has not been investigated in detail. In this study, novel CuBi_2_O_4_/*β*-Bi_2_O_3_ composite photocatalysts with different mass ratios and calcination temperatures were firstly synthesized by the hydrothermal method following a temperature-programmed process. The as-synthesized composites were systematically characterized and their photocatalytic performance was carefully investigated by the degradation of DS under visible-light irradiation (*λ* > 400 nm). Moreover, the active species in the CuBi_2_O_4_/*β*-Bi_2_O_3_ photocatalytic system were discussed through the free radical capture experiments, and the photocatalytic mechanism of CuBi_2_O_4_/*β*-Bi_2_O_3_ was also put forward.

## 2. Materials and Methods 

### 2.1. Materials

All reactants and solvents were analytical grade and used without further purification. Bi(NO_3_)_3_·5H_2_O, Cu(NO_3_)_2_·3H_2_O, Na_2_CO_3_, NaOH, HNO_3_, gluconic acid, tert-butanol (*t*-BuOH), para-benzoquinone (BZQ), disodium ethylenediaminetetr-aacetate (Na_2_-EDTA), ethanol, and diclofenac sodium were obtained from Sinopharm Chemical Reagent Co., Ltd., (Shanghai, China). Ultrapure water was used throughout this study.

### 2.2. Synthesis of CuBi_2_O_4_/β-Bi_2_O_3_

**Figure 1:**
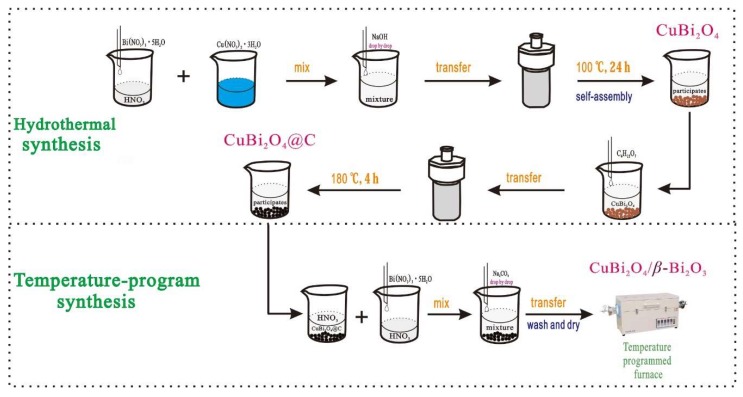
The detailed synthesis pathway of CuBi_2_O_4_/*β*-Bi_2_O_3_.

The detailed synthesis pathway of CuBi_2_O_4_/*β*-Bi_2_O_3_ is illustrated in Figure 1. Firstly, CuBi_2_O_4_ was prepared through a simple hydrothermal method, and the detailed experiment processes are similar to that reported by our previous paper [16]. Secondly, a core-shell structural CuBi_2_O_4_@C was synthesized, that is, 0.1 g of CuBi_2_O_4_ was ultrasonically dispersed into 70 mL of aqueous solution (with 0.3 mL gluconic acid), and then the solution was transferred into a 100-mL sealed Teflon-lined stainless steel autoclave for 4 h at 180 °C. When the autoclave was cooled naturally to room temperature, the precipitate was isolated by centrifugation and washed several times with distilled water. Thirdly, a temperature-programmed method was used to synthesize the CuBi_2_O_4_/*β*-Bi_2_O_3_. Then 0.1 g of CuBi_2_O_4_@C obtained above was ultrasonically dispersed into 20 mL HNO_3_ solution (1 mol/L) to obtain solution A; 0.39 mmol Bi(NO_3_)_3_·5H_2_O was completely dissolved into another 20 mL HNO_3_ solution (1 mol/L) under the acute agitation to obtain solution B; 2.34 mmol Na_2_CO_3_ was dissolved into 40 mL ultrapure water to obtain solution C. After solutions A and B were well mixed, solution C was added into the mixture drop by drop, and a large amount of white precipitate was produced. Then the washed precipitate by ethanol and ultrapure water was placed into a temperature-programmed furnace, and the reaction furnace was set to be heated to 600 °C within 30 min and kept at this temperature for 5 h. The CuBi_2_O_4_/*β*-Bi_2_O_3_ with mass ratio of 1:2.25 at different calcination temperatures of 400 °C, 600 °C and 800 °C was obtained through changing the temperature of the reaction furnace. In addition, the CuBi_2_O_4_/*β*-Bi_2_O_3_ with different mass ratios of 1:1, 1:2.25 and 1:4 was obtained through adjusting the added amount of Bi(NO_3_)_3_·5H_2_O and Na_2_CO_3_. For composition, the pure CuBi_2_O_4_ and *β*-Bi_2_O_3_ were also prepared through the above synthesis process, and the detailed synthesis steps can also be seen from the Figure 1.

### 2.3. Characterization 

Powder X-ray diffraction (XRD) patterns were examined to study the crystal structure and phase composition of the materials using the D/max 2500 PC (Rigaku, Japan) instrument with Cu K*α* radiation (40 kV, 100 mA) at a rate of 4.0°/min over a 2*θ* range of 20°–60°. Morphologies of the samples were characterized by scanning electron microscopy (SEM, JSM-6360LV, JEOL, Tokyo, Japan). Ultraviolet-visible (UV-Vis) diffusive reflectance spectra were obtained by a Shimadzu UV-2550 spectrophotometer (Shimadzu, Kyoto, Japan) over the analysis range from 200 to 800 nm. X-ray photoelectron spectra (XPS) were recorded using the ESCALAB 250 instrument (Thermo Scientific, Waltham, MA, USA) with Al K*α* radiation.

### 2.4. Photocatalytic Performance Experiments

The photocatalytic performance experiments were conducted in photoreaction apparatus (BL-GHX-V, Bilang Biological Science and Technology Co., Ltd., Xi’an, China) using a 300 W Xe lamp with an ultraviolet cutoff filter (providing visible light ≥ 400 nm) as the light source. In each experiment, a 25 mg photocatalyst was added to 50 mL of DS solution (8 mg/L). Before irradiation, the solution was magnetically stirred in the dark for 30 min to reach the adsorption-desorption equilibrium. As the reaction time elapsed, the sample was taken out and filtered immediately with 0.45 µm membrane filters, the concentrations of DS and Total Organic Carbon (TOC) are measured by a high performance liquid chromatography (HPLC, Agilent 1260, Santa Clara, CA, USA) and TOC (Shimadzu TOC-V_CPH_, Kyoto, Japan) analyzer, respectively. In addition, repeated experiments for DS degradation were also conducted to study the stability of the as-prepared photocatalysts, and the operation processes were similar to the photocatalytic experiments. All experiments were repeated twice and the data shown in the article was averaged.

### 2.5. Analysis of Reactive Species

Free radical capture experiments were used to ascertain the reactive species of CuBi_2_O_4_/*β*-Bi_2_O_3_ photocatalytic system, and tert-butanol (*t*-BuOH) was chosen as the hydroxyl radical (OH^•^) scavenger, disodium ethylenediamine tetraacetate (EDTA-Na_2_) was chosen as the hole (*h*^+^) scavenger, benzoquinone (BZQ) was chosen as the superoxide radical (O_2_^•−^) scavenger. The detailed experiment processes were similar to the photocatalytic activity experiments.

## 3. Results and Discussion

**Figure 2:**
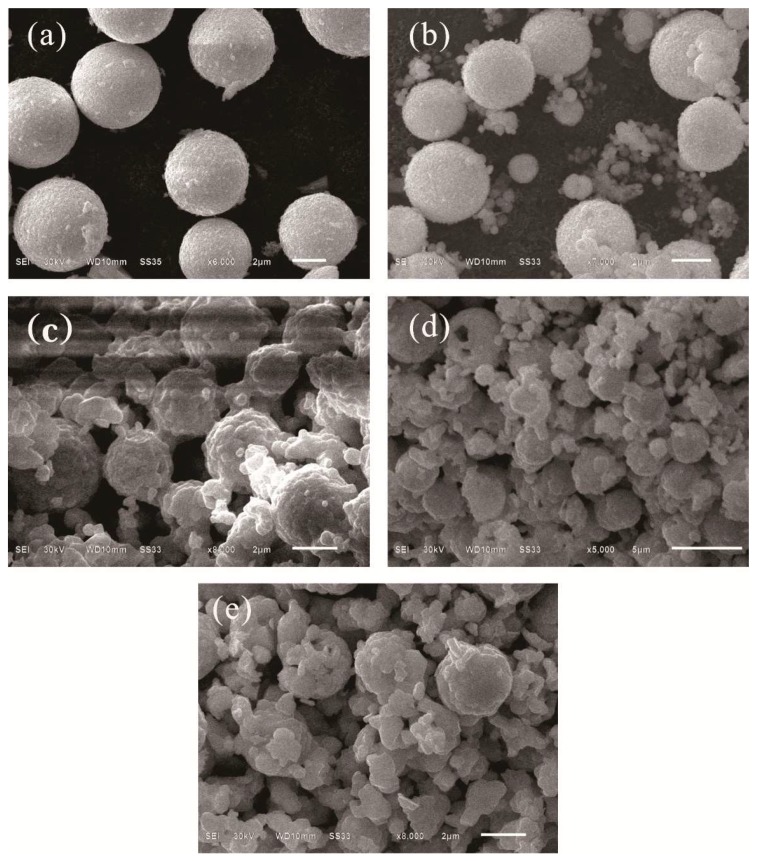
Scanning electron microscope (SEM) images of (**a**) CuBi_2_O_4_; (**b**) CuBi_2_O_4_@C; (**c**) CuBi_2_O_4_/*β*-Bi_2_O_3_ (1:2.25, 400 °C); (**d**) CuBi_2_O_4_/*β*-Bi_2_O_3_ (1:2.25, 600 °C); (**e**) CuBi_2_O_4_/*β*-Bi_2_O_3_ (1:2.25, 800 °C).

Figure 2 depicts scanning electron microscope (SEM) images of the as-prepared materials. A well-distributed micro-sphere structure (~5 μm) with smooth surface for pure CuBi_2_O_4_ is shown in Figure 2a. The transition material of CuBi_2_O_4_@C, from Figure 2b, shows increased size and relatively rough surface compared with pure CuBi_2_O_4_. Moreover, some dispersed carbon microspheres exist as well. In the formation process of CuBi_2_O_4_/*β*-Bi_2_O_3_, the control of temperature is an important step. As shown from Figure 2c, the products exhibit a chain-connected spherical structure when the temperature is at 400 °C, and the connection matter is the incompletely burned carbon. That is because the complete combustion temperature of carbon is higher than 500 °C [29]. Therefore, the composition of calcined products just contain CuBi_2_O_4_ and *β*-Bi_2_O_3_ when the temperatures are at 600 °C and 800 °C. But the rising of calcination temperature will destroy sphere-structural CuBi_2_O_4_, which can be seen from the SEM images of Figure 2d,e.

**Figure 3:**
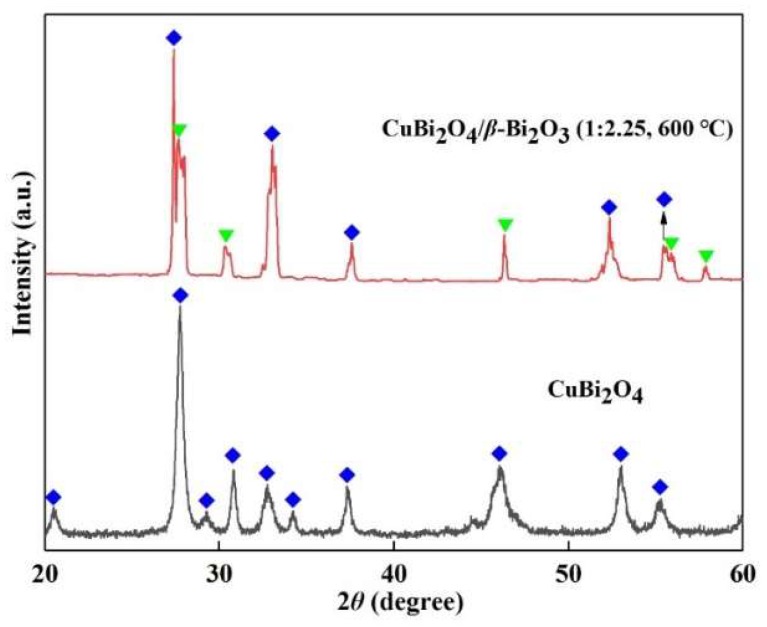
X-ray diffraction (XRD) patterns of CuBi_2_O_4_ and CuBi_2_O_4_/*β*-Bi_2_O_3_ (1:2.25, 600 °C).

The purity and crystallinity of CuBi_2_O_4_ and CuBi_2_O_4_/*β*-Bi_2_O_3_ calcined at 600 °C were examined with XRD, as depicted in Figure 3. From the XRD pattern of CuBi_2_O_4_, the diffraction peaks can be perfectly indexed to the phase of CuBi_2_O_4_ (JCPDS No. 84-1969) [30]. In the XRD pattern of CuBi_2_O_4_/*β*-Bi_2_O_3_ (600 °C), except for the peaks indicating CuBi_2_O_4_, the diffraction peaks located at the 2*θ* values of 27.66°, 32.86°, 46.32°, 55.44°, and 57.86°, can be indexed to the (201), (220), (222), (421), and (402) crystalline planes of tetragonal *β*-Bi_2_O_3_ (JCPDS No. 27-0050) [22,25]. Moreover, there are no obvious carbon peaks from the XRD pattern of CuBi_2_O_4_/*β*-Bi_2_O_3_ (600 °C), indicating the complete combustion of carbon in this calcination temperature [29].

**Figure 4:**
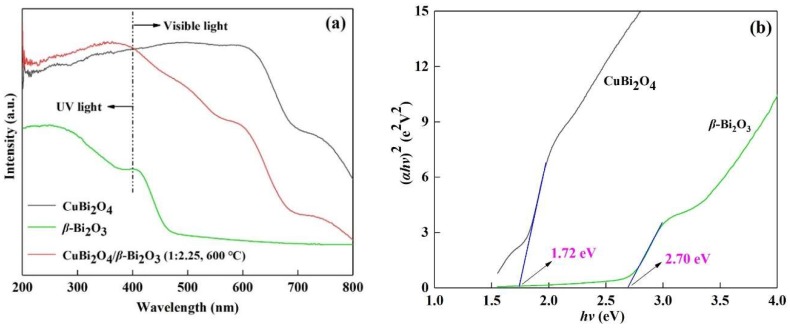
(**a**) Ultraviolet-visible (UV-Vis) diffuse reflectance spectra of CuBi_2_O_4_ and CuBi_2_O_4_/*β*-Bi_2_O_3_ (1:2.25, 600 °C); (**b**) band gap energy (*E*_g_) of CuBi_2_O_4_ and CuBi_2_O_4_/*β*-Bi_2_O_3_ (1:2.25, 600 °C) derived from the plots of (*αhv*)^2^ versus energy (*hv*).

The UV-Vis absorption spectrum of CuBi_2_O_4_, *β*-Bi_2_O_3_ (600 °C) and CuBi_2_O_4_/*β*-Bi_2_O_3_ (600 °C) are displayed in Figure 4a. Both of the materials exhibit strong absorbance in the UV and visible light regions, and the maximum absorption boundary of CuBi_2_O_4_, *β*-Bi_2_O_3_ (600 °C) and CuBi_2_O_4_/*β*-Bi_2_O_3_ (600 °C) appear at approximately 800 nm, 500 nm and 725 nm, respectively. The band gap energy (*E*_g_) of CuBi_2_O_4_ and *β*-Bi_2_O_3_ (600 °C) can be determined with the classic Tauc approach by using the following equation [31]: $\alpha hv=A{\left(hv-E_{g}\right)}^{n/2}$, where *α*, *h*, *v*, *E*_g_ and *A* are the absorption coefficient, the Planck constant, the light frequency, the band gap energy, and a constant, respectively. In the equation, *n* is a number characteristic of the charge transition in a semiconductor, and *n* = 1 for a direct transition while *n* = 4 for an indirect transition [31]. As for CuBi_2_O_4_ and *β*-Bi_2_O_3_ (600 °C), *n* = 4 [32,33]. Therefore, their band gap energy could be elicited from the plot of light energy (*αhv*)^2^ versus energy (*hv*), shown in Figure 4b, which suggests that the band gap energy of CuBi_2_O_4_ and *β*-Bi_2_O_3_ (600 °C) are 1.72 eV and 2.70 eV, respectively, which are very close to previous literature [33,34,35]. In addition, the valence band (VB) and conduction band (CB) edge position of the semiconductors can be estimated according to the following empirical equation:

*E*_VB_ = *X* − *E*^c^ + 0.5*E*_g_ (1)

(2) $$\;E_{\mathrm{CB}}=E_{\mathrm{VB}}-E_{g}\;$$

where *E*_VB_ and *E*_CB_ are the valence band (VB) and conduction band (CB) edge potentials, respectively; *X* is the electronegativity of the absolute electronegativity of the constituent atoms, that is 4.59 eV for CuBi_2_O_4_ and 6.24 eV for *β*-Bi_2_O_3_ [25,33]; *E*^c^ is the energy of free electrons on the hydrogen scale (approximately 4.5 eV) [31]. Consequently, the *E*_VB_ and *E*_CB_ positions of CuBi_2_O_4_ are estimated to be 0.95 and −0.77 eV/NHE; the *E*_VB_ and *E*_CB_ positions of *β*-Bi_2_O_3_ (600 °C) are estimated to be 3.09 and 0.39 eV/NHE.

**Figure 5:**
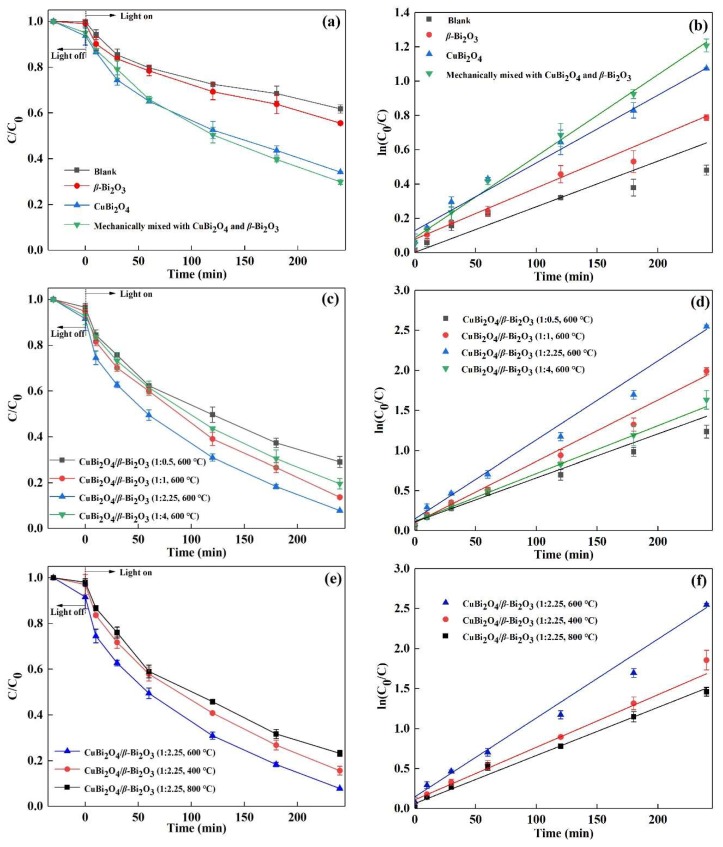
Diclofenac sodium (DS) degradation efficiency in the photocatalytic system of (**a**) blank irradiation, CuBi_2_O_4_, *β*-Bi_2_O_3_ and CuBi_2_O_4_ + *β*-Bi_2_O_3_; (**c**) CuBi_2_O_4_/*β*-Bi_2_O_3_ with different mass ratios; (**e**) CuBi_2_O_4_/*β*-Bi_2_O_3_ with different calcination temperatures; (**b**,**d**,**f**) plots of ln(C_0_/C) versus irradiation time over different photocatalytic systems.

The degradation of DS in different photocatalytic systems was evaluated under visible light irradiation, and the results are described inFigure 5. It can be seen from Figure 5a, the degradation efficiency of DS in the blank irradiation system is 38.21% in 240 min reaction while the degradation efficiency of DS is 44.48% and 65.85% with the addition of pure *β*-Bi_2_O_3_ and CuBi_2_O_4_. As for the system of mechanically mixed CuBi_2_O_4_ and *β*-Bi_2_O_3_ with the mass ratio of 1:2.25, 70.11% of DS can be degraded in the same condition. Figure 5c shows the photodegradation efficiency of DS in the system of composites CuBi_2_O_4_/*β*-Bi_2_O_3_ with different mass ratios. The adsorption capacity of composites to DS is less than 10%, suggesting the removal of DS is closely related to photodegradation and photocatalysis. The degradation efficiency of DS is enhanced with increasing *β*-Bi_2_O_3_ in the composite as the mass ratio of CuBi_2_O_4_ and *β*-Bi_2_O_3_ changes from 1:0.5 to 1:2.25, but will decrease with a further increase of the content of *β*-Bi_2_O_3_, i.e., the mass ratio of 1:4. Figure 5e shows the degradation efficiency of DS using CuBi_2_O_4_/*β*-Bi_2_O_3_ calcined at different temperatures. It can be seen that the degradation efficiencies of DS are 84.37%, 92.17% and 76.80%, respectively, and the composites calcined at 400 °C, 600 °C and 800 °C. Figure 5b,d,f describe the degradation rate curves of DS in different photocatalytic systems which derive from the ln(C_0_/C) versus irradiation time and the values of degradation rates are listed in Table 1. The maximum degradation rate of DS can be obtained in the system of composite CuBi_2_O_4_/*β*-Bi_2_O_3_ with the mass ratio of 1:2.25 and the calcination temperature of 600 °C, which is 0.0099 min^−1^.

**Figure 6:**
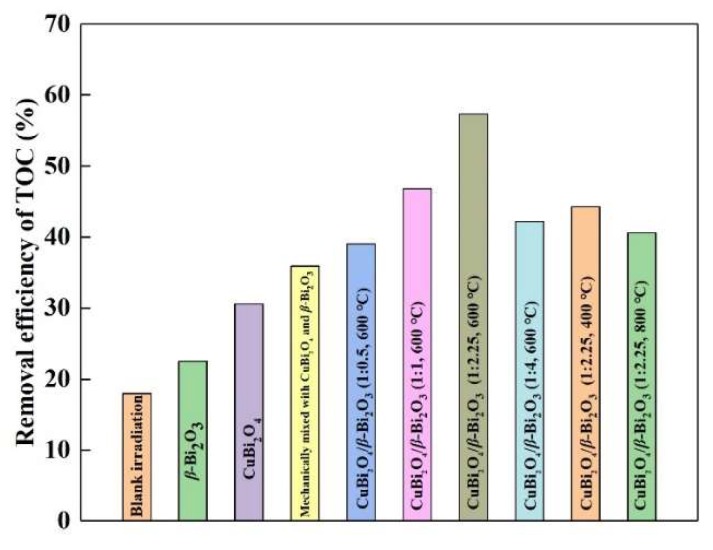
Total Organic Carbon (TOC) removal efficiency of the DS photodegradation solutions in different photocatalytic systems.

To further evaluate the degradation efficiency of DS in the as-prepared catalyst’s system, the removal efficiencies of Total Organic Carbon (TOC) are also explored and the results are shown in Figure 6. In the blank irradiation system, only 18.01% of TOC can be removed. And in the pure *β*-Bi_2_O_3_ and CuBi_2_O_4_ photocatalytic systems, the TOC removal efficiencies are 22.52% and 30.59%, respectively. When CuBi_2_O_4_ and *β*-Bi_2_O_3_ with the mass ratio of 1:2.25 are mechanically mixed to add into the system, 35.96% of TOC are removed. But for the composites CuBi_2_O_4_/*β*-Bi_2_O_3_ with different mass ratios calcinated at various temperatures, the removal efficiencies of TOC can be greatly improved, and the maximum value reaches 57.37%, which is achieved in the photocatalytic system of CuBi_2_O_4_/*β*-Bi_2_O_3_ with the mass ratio of 1:2.25 and the calcination temperature of 600 °C.

**Figure 7:**
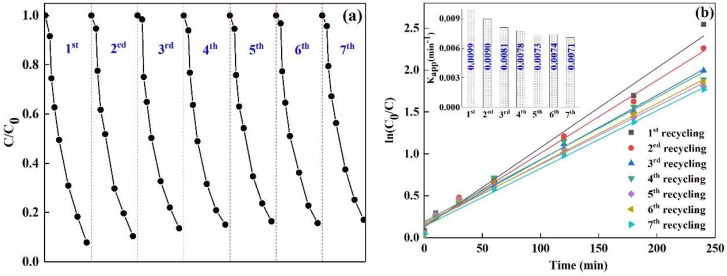
(**a**) Photodegradation efficiency of DS; (**b**) plots of ln(C_0_/C) versus irradiation time over CuBi_2_O_4_/*β*-Bi_2_O_3_ (1:2.25, 600 °C) under different recycling runs. The illustration in (**b**) shows the degradation rate constants of DS under different recycling runs.

**Figure 8:**
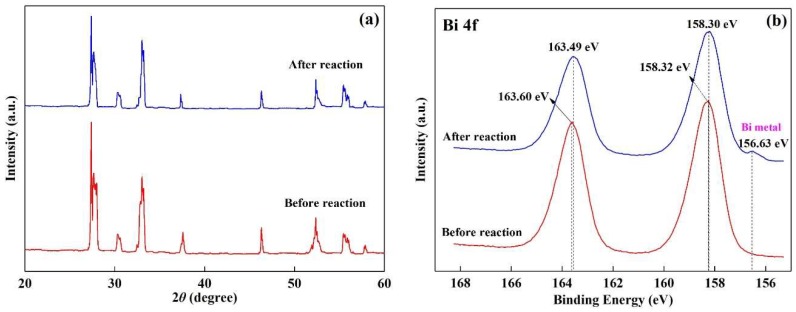
(**a**) XRD pattern; (**b**) Bi 4f X-ray photoelectron spectrum (XPS) of the composite catalyst CuBi_2_O_4_/*β*-Bi_2_O_3_ (1:2.25, 600 °C) before and after reaction for seventh times.

Figure 7a describes the photodegradation efficiency of DS under different recycling runs using CuBi_2_O_4_/*β*-Bi_2_O_3_ (1:2.25, 600 °C) as the catalyst. When the catalyst was repeated for seven times, the degradation efficiency of DS is 82.95%. Figure 7b shows the degradation rate curves of DS over CuBi_2_O_4_/*β*-Bi_2_O_3_ (1:2.25, 600 °C) under different recycling runs, and the values of rate constants are described in the illustration. It can be seen that the degradation rate of DS decreases from 0.0099 min^−1^ to 0.0071 min^−1^ after the seventh recycle. To further understand the photocatalytic stability of the as-prepared CuBi_2_O_4_/*β*-Bi_2_O_3_, XRD and Bi 4f XPS spectrums of the recycled composites are conducted seven times and the results are shown in Figure 8. Compared with the XRD spectra of the fresh and reused CuBi_2_O_4_/*β*-Bi_2_O_3_ in Figure 8a, there are no obvious changes for the main peaks. But from the Figure 8b, a new peak occur in the composite after recycling expect for the two main peaks existing in both of the two samples. According to reports, the main 4f 7/2 peak at 158.32 eV for a fresh sample and 158.30 eV for a reused sample, and the other main 4f 5/2 peak at 163.60 eV for a fresh sample and 163.49 eV for a reused sample, correspond to the Bi^3+^ oxidation state [36,37], which are in accordance to the presence of either CuBi_2_O_4_ or *β*-Bi_2_O_3_ phases. The extra peak at 156.63 eV in the reused sample is ascribed to Bi metal [38], indicating that some Bi^3+^ in the substance was reduced to be Bi metal after the reaction.

**Figure 9:**
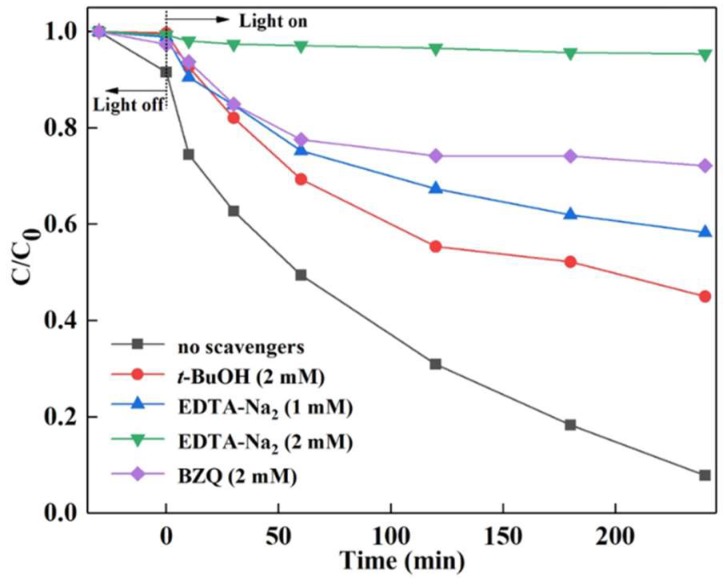
Photodegradation efficiency of DS under different scavengers in the system of CuBi_2_O_4_/*β*-Bi_2_O_3_ (1:2.25, 600 °C).

Moreover, the free radical capture experiments used to investigate the active species involved in the DS degradation with the CuBi_2_O_4_/*β*-Bi_2_O_3_ (1:2.25, 600 °C) photocatalyst are explored and the results are shown in Figure 9. The degradation efficiency of DS decreases to 41.74% when 1 mM EDTA-Na_2_ was added, and the DS degradation was completely inhibited with the addition of 2 mM EDTA-Na_2_. But the degradation efficiency of DS decreases to 54.98% and 27.82% when 2 mM *t*-BuOH or BZQ was added, respectively. The results suggest that the enhanced photocatalytic activity of CuBi_2_O_4_/*β*-Bi_2_O_3_ is closely related to OH^•^, *h*^+^ and O_2_^•−^, and the contribution order is *h*^+^ > O_2_^•−^ > OH^•^.

**Figure 10:**
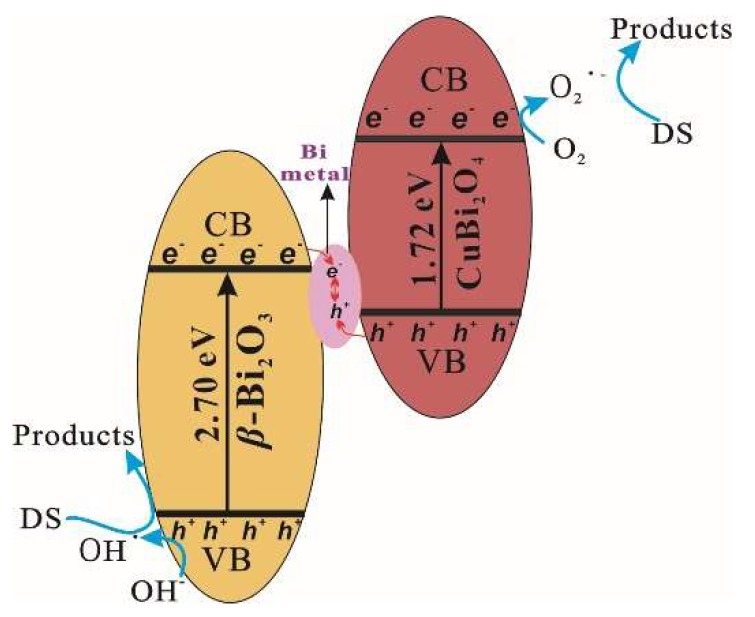
Supposed photocatalytic mechanism of CuBi_2_O_4_/*β*-Bi_2_O_3_ by the Z-scheme theory.

As for the photocatalytic mechanisms of a semiconductor–semiconductor composite catalyst, the heterojunction energy band theory and Z-scheme theory were of concern [8]. Based on the analysis above, a possible Z-scheme photocatalytic mechanism was put forward to explain the enhanced photocatalytic activity of the CuBi_2_O_4_/*β*-Bi_2_O_3_ composite, which is illustrated in Figure 10. That is, both CuBi_2_O_4_ and *β*-Bi_2_O_3_ could generate the photoinduced electron-hole pairs under visible-light illumination owing to their good light absorption abilities. Then, the formed Bi metal becomes the recombination center of the photogenerated electron from CB of *β*-Bi_2_O_3_ and the holes from the VB of the CuBi_2_O_4_ in the photocatalytic reaction process, leading to the improved charge separation. Besides, the dissolved O_2_ in the solution can be captured by the photogenerated electrons in the CB of CuBi_2_O_4_ to form O_2_^•−^ due to the more negative CB level of CuBi_2_O_4_ than the potential of O_2_/O_2_^•−^ (E(O_2_/O_2_^•−^) = 0.13 eV (vs. NHE) [39]; while the photogenerated holes in the VB of *β*-Bi_2_O_3_ can lead the H_2_O/OH^−^ to be oxidized to OH^•^ for the higher energy of holes in the VB of *β*-Bi_2_O_3_ than the potential of OH^−^/OH^•^ E(OH^−^/OH^•^) = 1.99 eV (vs. NHE) [39]. Subsequently, the holes in the VB of *β*-Bi_2_O_3_, the formed OH^•^ and O_2_^•−^ participate in the DS photodegradation.

## 4. Conclusions

A combined hydrothermal and temperature-programmed method was employed to synthesize the CuBi_2_O_4_/*β*-Bi_2_O_3_ composite photocatalysts. The properties of the as-prepared materials were systematically characterized by SEM, XRD, XPS and UV-Vis. Moreover, the composites exhibit enhanced photocatalytic performance toward DS degradation under visible light irradiation, and the optimal photodegradation efficiency of DS is achieved in the catalytic system of CuBi_2_O_4_/*β*-Bi_2_O_3_ with the mass ratio of 1:2.25 and the calcination temperature of 600 °C. Besides, the active species in the CuBi_2_O_4_/*β*-Bi_2_O_3_ photocatalytic system were discussed through the free radical capture experiments, and the photocatalytic mechanism of CuBi_2_O_4_/*β*-Bi_2_O_3_ was also put forward.

## Figures and Tables

**Table 1:** Kinetic analysis of DS degradation in different photocatalyst systems.

Photocatalytic System	K_app_ (min^−1^)	*R*^2^
Blank irradiation	0.0027	0.92
*β*-Bi_2_O_3_	0.0030	0.90
CuBi_2_O_4_	0.0040	0.94
Mechanically mixed with CuBi_2_O_4_ and *β*-Bi_2_O_3_	00048	0.94
CuBi_2_O_4_/*β*-Bi_2_O_3_ (1:0.5, 600 °C)	0.0055	0.92
CuBi_2_O_4_/*β*-Bi_2_O_3_ (1:1, 600 °C)	0.0076	0.91
CuBi_2_O_4_/*β*-Bi_2_O_3_ (1:2.25, 600 °C)	0.0099	0.90
CuBi_2_O_4_/*β*-Bi_2_O_3_ (1:4, 600 °C)	0.0059	0.96
CuBi_2_O_4_/*β*-Bi_2_O_3_ (1:2.25, 400 °C)	0.0065	0.95
CuBi_2_O_4_/*β*-Bi_2_O_3_ (1:2.25, 800 °C)	0.0060	0.95
